# Schwannoma Arising From the Common Palmar Digital Nerve of the Ulnar Nerve: A Report of a Rare Case

**DOI:** 10.7759/cureus.117771

**Published:** 2026-10-11

**Authors:** Abdelmounaim Daoudi, Ismail Dafali, Soufiane Bouziani, Walid Bouziane, Sadougui Med, Abdelkrim Daoudi

**Affiliations:** 1 Orthopedics and Traumatology, Centre Hospitalier Universitaire Mohammed VI d’Oujda, Oujda, MAR

**Keywords:** common palmar digital nerve, hand surgery, peripheral nerve surgeries, schwannoma, tumor

## Abstract

Schwannomas are benign tumors of the peripheral nerve sheath arising from Schwann cells and can affect the hand. However, involvement of the interdigital nerves remains rare. We report the case of a solitary schwannoma occurring in the common palmar digital nerve of the ulnar nerve in a 42-year-old man who presented with localized pain in the fourth palmar interdigital space and a small, slowly enlarging mass. The diagnosis was suggested by clinical examination and imaging (high-resolution ultrasound and MRI) and confirmed by histopathology following nerve-sparing marginal excision. The patient experienced immediate relief of symptoms, retained sensation in the fingers, and had no recurrence at 12 months. This case highlights the importance of considering schwannoma in the differential diagnosis of interdigital masses of the hand, the role of targeted imaging in surgical planning, and the effectiveness of nerve-sparing microsurgical excision.

## Introduction

Schwannomas (neurilemomas) are benign, encapsulated tumors arising from Schwann cells that surround peripheral nerve fibers [1]. While they account for less than 5% of all benign soft tissue tumors [1,2], the upper extremity is a common site of presentation, representing 19-47% of cases [2,3]. However, involvement of the hand is relatively uncommon, comprising only 7-10% of upper limb schwannomas [4,5], and interdigital localization remains exceptionally rare [6-9].

Because interdigital schwannomas are often small and present with nonspecific symptoms that mimic ganglion cysts or lipomas, preoperative diagnosis is frequently delayed [2,10,11]. Furthermore, lesions originating specifically from the common palmar digital nerve of the ulnar nerve are extremely unusual, as most hand cases affect the median nerve [12,13]. Accurate preoperative identification using high-resolution ultrasound or MRI facilitates nerve-sparing enucleation and optimizes functional outcomes [11,14,15].

## Case presentation

A 42-year-old man presented with a 10-month history of intermittent, throbbing pain localized to the palmar interdigital space between the fourth and fifth fingers of his right hand. His symptoms were exacerbated by manual labor. Four months before his visit, he had noticed a small mass that was slowly growing in this palmar interdigital space. There was no history of trauma, systemic disease, or previous surgery involving the hand.

Physical examination revealed a firm, well-defined subcutaneous mass measuring approximately 2 cm in maximum diameter. The mass was slightly mobile and tender to deep palpation. Tinel’s sign at the site of the lesion reproduced the pain. No sensory deficits or objective motor dysfunction were identified in the fingers.

Standard radiographs of the hand revealed no bony abnormalities or soft-tissue radiopacities (Figure 1).

**Figure 1:**
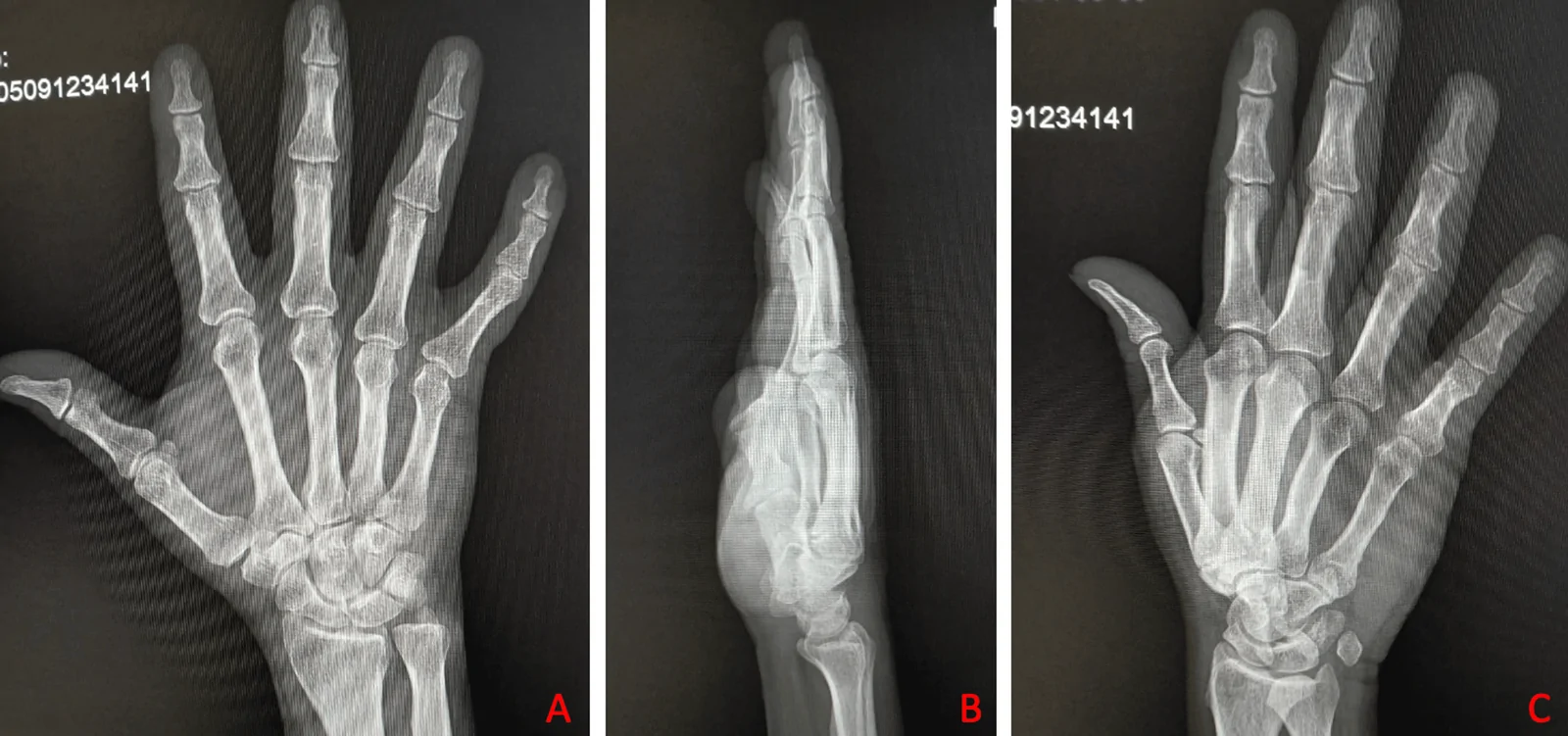
Standard radiographs of the right hand. (A) Anteroposterior, (B) lateral, and (C) oblique views demonstrating normal bone architecture and joint alignment, with no signs of cortical erosion, periosteal reaction, or abnormal soft-tissue radiopacities.

High-resolution ultrasound revealed a hypoechoic, ovoid, well-defined lesion measuring 2.1 × 0.9 × 0.8 cm on the palmar aspect of the fourth interdigital space of the right hand, with posterior acoustic enhancement and internal vascularization. The lesion appeared displaced relative to the common palmar digital nerve of the ulnar nerve.

MRI of the hand with contrast revealed a well-defined, encapsulated mass on the palmar side, adjacent to the common palmar digital nerve of the ulnar nerve. The mass was isointense relative to muscle on T1-weighted images and hyperintense on T2-weighted images, with homogeneous enhancement following contrast administration and a fascicular appearance suggestive of a nerve sheath tumor (Figure 2). MRI is useful for defining the relationship between a peripheral nerve tumor and its parent nerve and for surgical planning [6].

**Figure 2:**
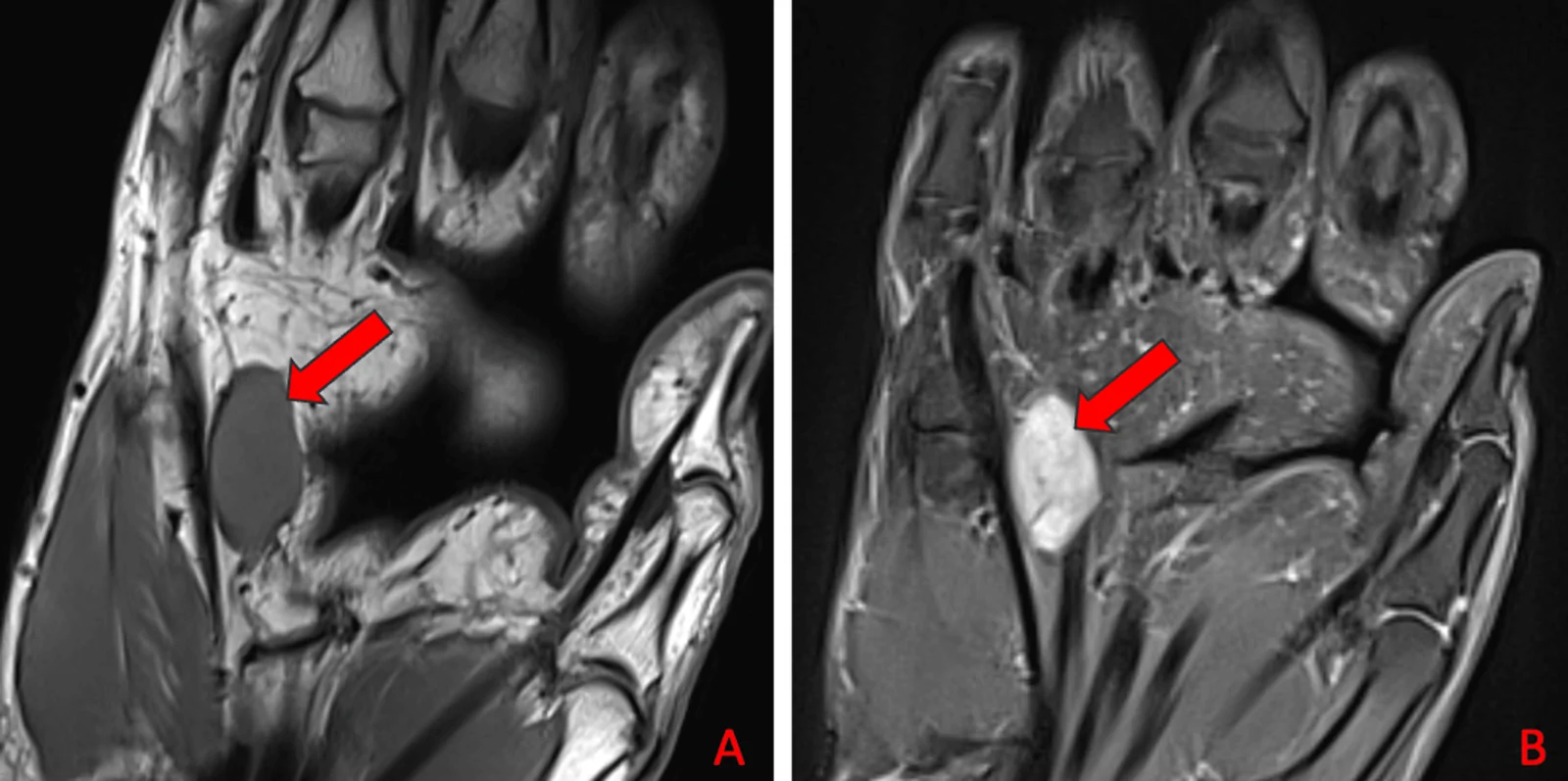
MRI of the right hand. (A) Coronal T1-weighted image revealing a well-circumscribed, encapsulated ovoid mass in the fourth palmar interdigital space, appearing isointense to hypointense relative to adjacent muscle tissue. (B) Coronal fat-suppressed image demonstrating the hyperintensity of the lesion, clearly delineating its relationship with the neurovascular bundle and showing features typical of a benign peripheral nerve sheath tumor.

Based on the clinical and radiological findings, surgery was planned.

The patient underwent surgery under regional anesthesia of the hand, using a magnifying loupe. A tourniquet was applied to the arm and inflated to 250 mmHg for 26 minutes. A longitudinal palmar incision was made at the site of the mass. Microsurgical dissection revealed a yellowish, encapsulated tumor located eccentrically relative to the common palmar digital nerve of the ulnar nerve (Figure 3A). Intracapsular marginal excision was performed, taking care to preserve the surrounding nerve bundles (Figure 3B). The specimen was sent for histopathological examination. The skin was closed with simple sutures. Nerve-sparing enucleation is the preferred surgical approach for symptomatic benign peripheral nerve schwannomas when the tumor can be separated from the functional nerve fascicles.

**Figure 3:**
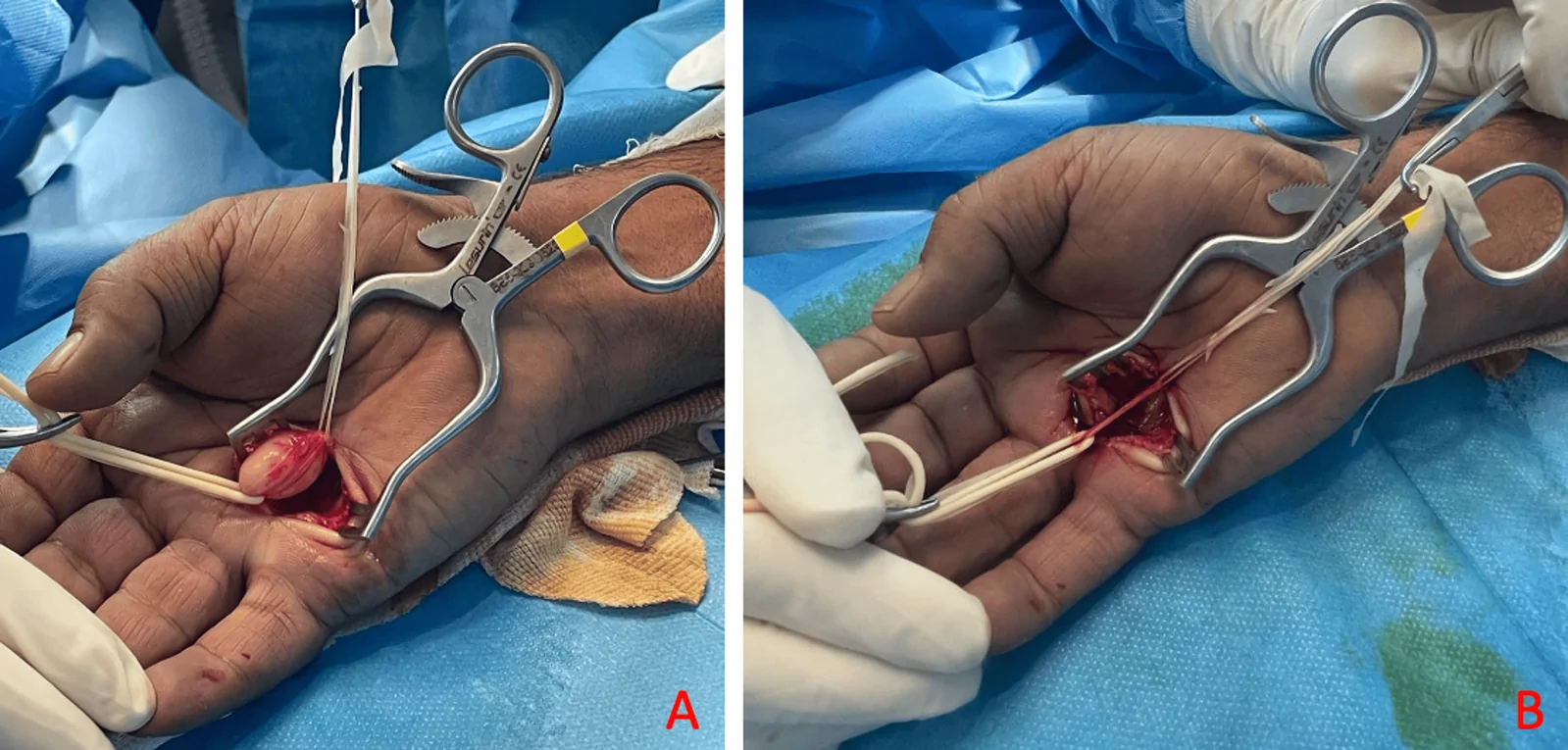
Intraoperative views. (A) Surgical dissection through a longitudinal palmar incision at the fourth interdigital space. The well-encapsulated, yellowish-pink tumor is isolated using vessel loops, demonstrating its eccentric location relative to the common palmar digital nerve of the ulnar nerve and allowing for nerve-sparing marginal enucleation. (B) Surgical site following complete tumor enucleation. The common palmar digital nerve of the ulnar nerve and its digital branches are clearly isolated on vessel loops, demonstrating structural continuity and preservation of the nerve architecture.

Histopathological examination revealed an encapsulated spindle-cell tumor with Verocay bodies (Figure 4A). Immunohistochemistry showed strong, diffuse positivity for S-100 protein (Figure 4B). No mitotic figures or cellular atypia were observed, confirming the diagnosis of a benign schwannoma.

**Figure 4:**
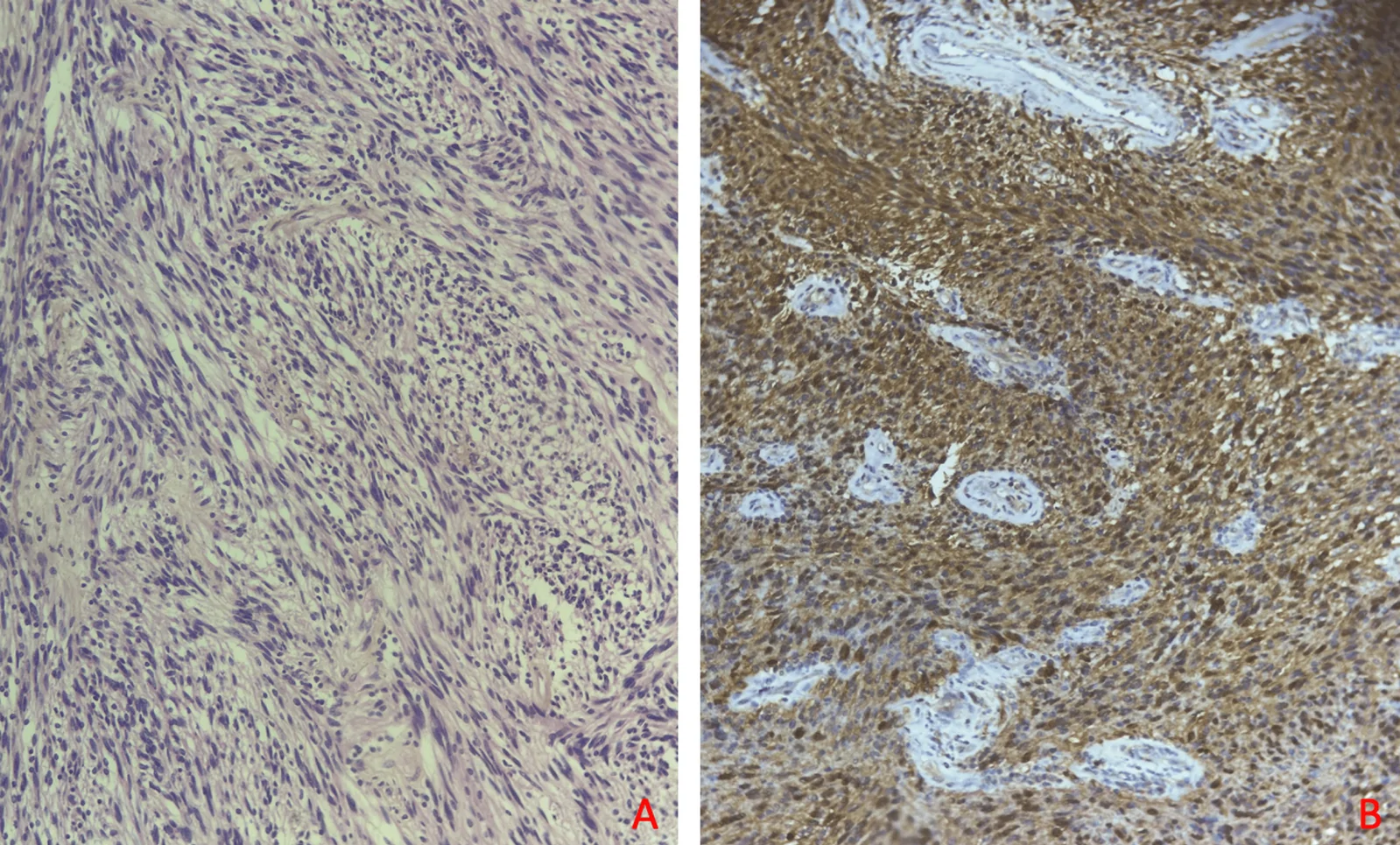
Histopathological and immunohistochemical findings. (A) Hematoxylin and eosin staining showing a highly cellular Antoni A pattern with prominent nuclear palisading around acellular eosinophilic zones, forming classic Verocay bodies. (B) Immunohistochemical analysis demonstrating strong, diffuse cytoplasmic and nuclear positivity for S-100 protein, confirming the diagnosis of a benign schwannoma.

The patient reported immediate relief from pressure-related pain after the operation and retained intact sensation in his fingers. The surgical wound healed without complications. The patient was followed up for 12 months, during which no signs of recurrence were observed.

## Discussion

Schwannomas are benign peripheral nerve sheath tumors that most commonly occur in adults, with a peak incidence during the third to sixth decades of life [1,2]. They are generally solitary and slow-growing [2]. Schwannomas of the upper extremity may involve major peripheral nerves, whereas involvement of small digital or interdigital nerves is considerably less frequent [7,10].

Involvement of the interdigital spaces of the hand is rare and may contribute to diagnostic delay [7,10]. The typical presentation consists of a slow-growing, well-circumscribed mass that may be tender to palpation and can be associated with paresthesia or a positive Tinel’s sign [6,7]. Motor deficits are uncommon because schwannomas generally displace rather than infiltrate the nerve fascicles [3,14].

Interdigital masses of the hand have a broad differential diagnosis, including ganglion cysts, giant cell tumors of the tendon sheath, vascular lesions, glomus tumors, lipomas, and other benign soft-tissue tumors [2,4]. Clinical assessment should consider the location, consistency, mobility, tenderness, and relationship of the lesion to the neurovascular structures. A detailed clinical examination combined with targeted imaging can therefore reduce diagnostic uncertainty and facilitate appropriate management [4,6].

High-resolution ultrasound is a useful first-line imaging modality for small peripheral nerve lesions [4,11]. Schwannomas generally appear as well-defined, hypoechoic, ovoid, or fusiform masses associated with a peripheral nerve [4,11]. Posterior acoustic enhancement and internal vascularity may also be observed [11]. Ultrasound can help establish the relationship between the lesion and the nerve, supporting the diagnosis of a peripheral nerve sheath tumor and facilitating nerve-sparing surgical planning [6,11].

MRI provides superior soft-tissue contrast and is particularly useful for characterizing peripheral nerve sheath tumors and defining their relationship with adjacent anatomical structures and parent nerves for surgical planning [5,11]. Typical MRI findings include a well-defined lesion that is iso- or hypointense relative to muscle on T1-weighted images and hyperintense on T2-weighted images, with enhancement after contrast administration [5,11]. A fascicular or target-like appearance may further support the diagnosis of a peripheral nerve sheath tumor [11]. In the present case, MRI clearly demonstrated the close anatomical relationship between the mass and the common palmar digital nerve [5].

Although imaging may strongly suggest a schwannoma, histopathological examination remains essential for definitive diagnosis [1,15]. The diagnosis is supported by characteristic spindle-cell morphology, Antoni A and Antoni B areas, Verocay bodies, and strong S-100 protein expression [1,15]. In the present case, the presence of Verocay bodies and diffuse S-100 positivity, in the absence of mitotic figures or cellular atypia, confirmed the diagnosis of a benign schwannoma [1,15].

The surgical goal is complete tumor excision while preserving nerve function [3,8,14]. For small, encapsulated schwannomas of the hand, intracapsular or marginal enucleation under magnification is the preferred surgical approach when the tumor can be separated from the functional nerve fascicles [3,8,14]. The surgical technique should include careful identification of the longitudinal axis of the nerve, exposure of the tumor capsule, identification of a safe capsular plane, and minimal traction on the surrounding nerve fascicles [3,14].

The present case illustrates the importance of identifying a plane between the tumor capsule and the functional nerve fascicles [3,14]. The tumor was eccentrically located relative to the common palmar digital nerve, allowing intracapsular marginal excision while preserving the surrounding fascicles [3,8]. Similar nerve-sparing approaches have been associated with favorable neurological outcomes after excision of benign peripheral nerve schwannomas [3,8,14].

When the tumor cannot be safely separated from the nerve fascicles, partial resection or nerve reconstruction may occasionally be considered, depending on the extent of involvement, the functional importance of the nerve, and the expected morbidity of nerve sacrifice [3,14]. Therefore, the surgical strategy should be individualized according to the anatomical relationship between the tumor and the nerve [3,14].

Malignant transformation of a solitary schwannoma is exceedingly uncommon [1,15]. Nevertheless, atypical histological findings, rapid growth, marked cellularity, necrosis, or increased mitotic activity should prompt further investigation and closer follow-up [1,15]. MRI may also contribute to the assessment of lesions with atypical clinical or radiological characteristics [11].

Postoperative neurological complications are generally uncommon when microsurgical, nerve-sparing techniques are used [3,8,14]. Transient sensory disturbances may occur, particularly as a result of manipulation or traction of the nerve fascicles, whereas persistent sensory loss or neuropathic pain is less frequent [3,14]. Large surgical series have demonstrated generally favorable neurological outcomes following excision of benign peripheral nerve schwannomas [3,9,12,14].

Recurrence following complete excision of a solitary schwannoma is uncommon [9,10]. The risk may be higher following incomplete or subtotal excision [9,10]. In a large series of 234 benign solitary schwannomas, only two recurrences were observed after apparently adequate excision [14]. Similarly, contemporary series of upper-extremity schwannomas have reported favorable outcomes and low recurrence rates after complete tumor excision [9,12,13].

A clinical follow-up strategy should be individualized according to the completeness of excision, pathological characteristics, anatomical location, and postoperative neurological status [9,11,13]. Additional ultrasound or MRI may be reserved for patients presenting with new symptoms, suspected recurrence, or atypical pathological findings [9,11].

In the present case, complete nerve-sparing excision resulted in immediate relief of the patient’s pain, preservation of digital sensation, uncomplicated wound healing, and no evidence of recurrence at 12 months. These findings are consistent with the generally favorable functional outcomes reported following surgical excision of benign peripheral nerve schwannomas [3,8,9,12-14].

## Conclusions

This case demonstrates that a structured diagnostic approach combining high-resolution imaging with microsurgical techniques is key to managing rare interdigital nerve sheath tumors. Schwannoma should be considered in the differential diagnosis of a painful, slowly growing interdigital mass, even in the absence of neurological deficits.

From a surgical standpoint, achieving a successful functional outcome relies on careful identification of the relationship between the tumor and the nerve fascicles and on the use of a safe capsular window for tumor enucleation. This meticulous nerve-sparing approach allows complete tumor removal while preserving digital sensation and minimizing neurological morbidity. Complete excision is associated with a low risk of recurrence, although appropriate clinical follow-up remains advisable.
